# Explainability for artificial intelligence in healthcare: a multidisciplinary perspective

**DOI:** 10.1186/s12911-020-01332-6

**Published:** 2020-11-30

**Authors:** Julia Amann, Alessandro Blasimme, Effy Vayena, Dietmar Frey, Vince I. Madai, the Precise4Q consortium

**Affiliations:** 1 Health Ethics and Policy Lab, Department of Health Sciences and Technology, ETH Zurich, Hottingerstrasse 10, 8092 Zurich, Switzerland; 2 Charité Lab for Artificial Intelligence in Medicine—CLAIM, Charité - Universitätsmedizin Berlin, Berlin, Germany; 3 School of Computing and Digital Technology, Faculty of Computing, Engineering and the Built Environment, Birmingham City University, Birmingham, UK

**Keywords:** Artificial intelligence, Machine learning, Explainability, Interpretability, Clinical decision support

## Abstract

**Background:**

Explainability is one of the most heavily debated topics when it comes to the application of artificial intelligence (AI) in healthcare. Even though AI-driven systems have been shown to outperform humans in certain analytical tasks, the lack of explainability continues to spark criticism. Yet, explainability is not a purely technological issue, instead it invokes a host of medical, legal, ethical, and societal questions that require thorough exploration. This paper provides a comprehensive assessment of the role of explainability in medical AI and makes an ethical evaluation of what explainability means for the adoption of AI-driven tools into clinical practice.

**Methods:**

Taking AI-based clinical decision support systems as a case in point, we adopted a multidisciplinary approach to analyze the relevance of explainability for medical AI from the technological, legal, medical, and patient perspectives. Drawing on the findings of this conceptual analysis, we then conducted an ethical assessment using the “Principles of Biomedical Ethics” by Beauchamp and Childress (autonomy, beneficence, nonmaleficence, and justice) as an analytical framework to determine the need for explainability in medical AI.

**Results:**

Each of the domains highlights a different set of core considerations and values that are relevant for understanding the role of explainability in clinical practice. From the technological point of view, explainability has to be considered both in terms how it can be achieved and what is beneficial from a development perspective. When looking at the legal perspective we identified informed consent, certification and approval as medical devices, and liability as core touchpoints for explainability. Both the medical and patient perspectives emphasize the importance of considering the interplay between human actors and medical AI. We conclude that omitting explainability in clinical decision support systems poses a threat to core ethical values in medicine and may have detrimental consequences for individual and public health.

**Conclusions:**

To ensure that medical AI lives up to its promises, there is a need to sensitize developers, healthcare professionals, and legislators to the challenges and limitations of opaque algorithms in medical AI and to foster multidisciplinary collaboration moving forward.

## Background

All over the world, healthcare costs are skyrocketing. Increasing life expectancy, soaring rates of chronic diseases, and the continuous development of costly new therapies contribute to this trend. Thus, it comes as no surprise that scholars predict a grim future for the sustainability of healthcare systems throughout the world. Artificial intelligence (AI) promises to alleviate the impact of these developments by improving healthcare and making it more cost-effective [1]. In clinical practice, AI often comes in the form of clinical decision support systems (CDSS), assisting clinicians in diagnosis of disease and treatment decisions. Where conventional CDSS match the characteristics of individual patients to an existing knowledge base, AI-based CDSSs apply AI models trained on data from patients matching the use-case at hand. Yet, despite its undeniable potential, AI is not a universal solution. As history has shown, technological progress always goes hand in hand with novel questions and significant challenges. Some of these challenges are tied to the technical properties of AI, others relate to the legal, medical, and patient perspectives, making it necessary to adopt a multidisciplinary perspective.

In this paper, we take such a multidisciplinary view on a major medical AI challenge: explainability. In its essence, explainability can be understood as a characteristic of an AI-driven system allowing a person to reconstruct why a certain AI came up with the presented predictions. An important point to note here is that explainability has many facets and, unfortunately, the terminology of explainability is not well defined. Other terms such as interpretability and/or transparency are often used synonymously [2, 3]. We thus simply refer to explainability or explainable AI throughout the manuscript and add the necessary context for understanding.

Explainability is a heavily debated topic with far-reaching implications that extend beyond the technical properties of AI. Even though research indicates that AI algorithms can outperform humans in certain analytical tasks (e.g. pattern recognition in imaging), the lack of explainability has been criticized in the medical domain [4]. Legal and ethical uncertainties surrounding this issue may impede progress and prevent novel technologies from fulfilling their potential to improve patient and population health. Yet, without thorough consideration of the role of explainability in medical AI, these technologies may forgo core ethical and professional principles, disregard regulatory issues, and cause considerable harm [5].

To contribute to the discourse on explainable AI in medicine, this paper seeks to draw attention to the interdisciplinary nature of explainability and its implications for the future of healthcare. In particular, our work focuses on the relevance of explainability for CDSS. The originality of our work lies in the fact that we look at explainability from multiple perspectives that are often regarded as independent and separable from each other. This paper has two central aims: (1) to provide a comprehensive assessment of the role of explainability in CDSS for use in clinical practice and; (2) to make an ethical evaluation of what explainability means for the adoption of AI-driven tools into clinical practice.

## Methods

Taking AI-based CDSS as a case in point, we discuss the relevance of explainability for medical AI from the technological, legal, medical, and patient perspective. To this end, we performed a conceptual analysis of the pertinent literature on explainable AI in these domains. In our analysis, we aimed to identify aspects relevant to determining the necessity and role of explainability for each domain, respectively. Drawing on these different perspectives, we then conclude by distilling the ethical implications of explainability for the future use of AI in the healthcare setting. We do the latter by examining explainability against the four ethical principles of autonomy, beneficence, non-maleficence, and justice.

## Results

### The technological perspective

From the technological perspective, we will explore two issues. First, what explainability methods are and, second, where they are applied in medical AI development.

With regards to methodology, explainability can either be an inherent characteristic of an algorithm or can be approximated by other methods [2]. The latter is highly important for methods that have until recently been labeled as “black-box models” such as artificial neural network (ANN) models. To explain their predictions, however, numerous methods exist today [6]. Importantly, however, inherent explainability will, in general, be more accurate than methods that only approximate explainability [2]. This can be attributed to the complex characteristics of many modern machine learning methods. In ANNs, for example, the inner workings of sometimes millions of weights between artificial neurons need to be interpreted in a way that humans can understand. Thus, contrasting methods with inherent explainability have a crucial advantage. However, these methods are usually also traditional methods, such as linear or logistic regression. For many use cases, there is an inferiority of these traditional methods in performance compared to modern state-of-the-art methods such as ANNs [7]. Thus, there is a trade-off between performance and explainability, and this trade-off is a big challenge for the developers of clinical decision support systems. It should be noted that some assume that this trade-off does not exist in reality, but it is a mere artifact of suboptimal modelling approaches, as pointed out by Rudin et al. [2]. While the work of Rudin et al. is important to raise attention to the shortcomings of approximating explainability methods, it is likely that some approximating methods, in contrast to the notion of [2], have value given the complex nature of explaining machine learning models. Additionally, while we can make the qualitative assessment that inherent explainability is likely better than approximated explainability, there exist only exploratory initial attempts to rank explainability methods quantitatively [8]. Notwithstanding, for many applications—and generally in AI product development—there is a de facto preference for modern algorithms such as ANNs. Additionally, it cannot be ruled out that for some applications such modern methods do exhibit actual higher performance. This necessitates to critically assess explainability methods further, both with regards to technical development, e.g. for methods ranking and optimization of methods for certain inputs, and with regards to the role of explainability from a multiple stakeholder view as done in the current work.

From the development point-of-view, explainability will regularly be helpful for developers to sanity check their AI models beyond mere performance. For example, it is highly beneficial to rule out that the prediction performance is based on meta-data rather than the data itself. A famous non-medical example was the classification task to discern between huskies and wolves, where the prediction was solely driven by the identification of a snowy background rather than real differences between huskies and wolves [9]. This phenomenon is also called a “Clever Hans” phenomenon [10]. Clever Hans phenomena are also found in medicine. An example is the model developed by researchers from Mount Sinai hospital which performed very well in distinguishing high-risk patients from non-high-risk patients based on x-ray imaging. However, when the tool was applied outside of Mount Sinai, the performance plummeted. As it turned out the AI model did not learn clinically relevant information from the images. In analogy to the snowy background in the example introduced above, the prediction was based on hardware related meta-data tied to the specific x-ray machine that was used to image the high-risk ICU patients exclusively at Mount Sinai [11]. Thus, the system was able to distinguish only which machine was used for imaging and not the risk of the patients. Explainability methods allow developers to identify these types of errors before AI tools go into clinical validation and the certification process, as the Clever Hans predictors (snowy background, hardware information) would be identified as prediction relevant by the explainability methods rather than meaningful features from a domain perspective. This saves time and development costs. It should be noted that explainability methods aimed at developers to provide insight into their models have different prerequisites than systems aimed at technologically unsavvy end-users such as clinical doctors and patients. For developers, these methods can be more complex in their approach and visualization.

### The legal perspective

From the legal perspective, the question arises if and, if yes, to what extent explainability in AI is legally required. Taking the cue from other fields such as public administration, transparency and traceability have to meet even higher standards when it comes to health care and the individual patient [12]. As shown above, artificial intelligence approaches such as machine learning and deep learning have the potential to significantly advance the quality of health care. Identifying patterns in diagnostics, anomaly detection and, in the end, providing decision support are already changing standards of care and clinical practice. To fully exploit these opportunities for improving patients’ outcomes and saving lives by advancing detection, prevention, and treatment of diseases, the sensitive issues of data privacy and security, patient consent, and autonomy have to be fully considered. This means that from a legal perspective, data—its acquisition, storage, transfer, processing, and analysis—will have to comply with all laws, regulations and further legal requirements. In addition, the law and its interpretation and implementation have to constantly adapt to the evolving state-of-the-art in technology [13]. Even when fulfilling all of these rather obvious requirements the question remains if the application of AI-driven solutions and tools demand explainability. In other words, do doctors and patients need information not only about the results that are provided but also about the characteristics and features these results are based upon, and the respective underlying assumptions. And, might the necessary inclusion of other stakeholders require an understanding and explainability of algorithms and models.

From a Western legal point-of-view, we identified three core fields for explainability: (1) Informed consent, (2) Certification and approval as medical devices (acc. to Food and Drug Administration/FDA and Medical Device Regulation/MDR) and (3) Liability.

Personal health data may be only processed by law after the individual consents to its use. In the absence of general laws facilitating the use of personal data and information, this informed consent is the standard for today’s use of patient data in AI applications [14]. This is particularly challenging since the consent has to be specified in advance, i.e. the purpose of the given project and its aims have to be outlined. The natural advantage of AI is that it does not necessitate pre-selection of features and can identify novel patterns or find new biomarkers. If restricted to specific purposes—as required for informed consent—this unique advantage might not be fully exploitable. For obtaining informed consent for diagnostic procedures or interventions the law requires individual and comprehensive information about and understanding of these processes. In the case of AI-based decision support, the underlying processes and algorithms have therefore to be explained to the individual patient. Just like in the case of obtaining consent for undergoing an MR imaging procedure, the patient might not necessarily need to know every detail but certainly has to be informed about core principles, and especially the risks. Yet, contrary to an MR imaging procedure, physicians are unable to provide this type of information for an opaque CDSS. What physicians should at least be able to provide are explanations around two principles: (1) the agent view of AI, i.e. what it takes as input; what it does with the environment; and what it produces as output, and (2) explaining the training of the mapping which produces the output by letting it learn from examples—which encompasses unsupervised, supervised, and reinforcement learning. Yet, it is important to note that for AI-based CDSS the extent of the information is a priori highly difficult to define, has to be adjusted to the respective use case, and will certainly need clarification from the legislative bodies. For this, a framework for defining the "right" level of explainability, as Beaudouin et al. put it [15], should be developed. Clearly, this also raises important questions about the role and tasks of physicians, underscoring the need for tailored training and professional development in the area of medical AI.

With regard to certification and approval as medical devices, the respective bodies have been slow to introduce requirements for explainable AI and its implications on the development and marketing of products. In a recent discussion paper, the FDA facilitates in its total product lifecycle approach (TPLC) the constant development and improvement of AI-based medical products. Explainability is not mentioned but an “Appropriate level of transparency (clarity) of the output and the algorithm aimed at users” is required [16]. This is mainly aimed at the functions of the software and its modifications over time. The MDR does not specifically regulate the need for explainability with regard to medical devices that use artificial intelligence and machine learning in particular. However, also here, the need for accountability and transparency are set and the evolution of xAI might lead the legislative and the notified bodies to change the regulations and their interpretation accordingly.

In conclusion, both FDA and MDR are currently rather vaguely requiring explainability, i.e. information for traceability, transparency, and explainability of development of ML/DL models that inform medical treatment. Most certainly, these requirements will be defined more precisely in the future mandating producers of AI-based medical devices/software to provide insight into the training and testing of the models, the data, and the overall development processes. We would also like to mention that there is a current debate on whether the General Data Protection Regulation (GDPR) in the European Union requires the use of explainable AI in tools working with patient data [17, 18]. Also here, it cannot be ruled out that the currently ambiguous phrasings will be amended in favor of one that promotes explainability in the future.

Finally, the question arises, to what extent the patient has to be made aware that treatment decisions such as those derived by a clinical decision support system might rely on AI and the legal and litigation question if the physician adhered to the recommendation or overruled the machine. For the US, as Cohen laid out, there is currently no clear-cut answer to what extent the integration of ML/DL into clinical decision-making has to be disclosed with regard to liability [14]. Hacker et al. argue that legally it is likely that explainability will be a prerequisite from a contract and tort law perspective where doctors may have to use a certain tool to avoid the threat of a medical malpractice lawsuit [17]. The final answer to this lies with the courts, however, and will be given rather sooner than later as an increasing number of AI-based systems will be in use.

Taken together, the legal implications of introducing AI technologies into health care are significant and the constant conflict between innovation and regulation needs careful orchestration. Potentially life-saving just as new cancer medication or antibiotics, AI-based decision support needs guidelines and legal crash barriers to avoid existential infringement on patients’ rights and autonomy. Explainability is an essential quality in this context and we would argue that performance is only sufficient in cases, where it is not possible to provide explainability. Overall, there is a strong need for explainability in legal aspects and opening the black box has become essential and will prove to be the watershed moment for the application of AI in medicine.

### The medical perspective

From the medical perspective, the first consideration is what distinguishes AI-based clinical decision support from established diagnostic tools, such as advanced laboratory testing for example? Especially as they do exhibit considerable overlaps: Both can provide results used for CDSSs, for both performance is a key issue, and their results are documentable. We also understand the inner working of laboratory testing, as it is often the case with other diagnostic tests, such as imaging, so they would not be regarded as black box methods. On the other hand, for these methods we cannot explain the result of any individual test. This makes it evident that from a medical perspective, we need to distinguish two levels of explainability. First level explainability allows us to understand how the system arrives at conclusions in general. In analogy to laboratory testing, where we know which biological and biochemical reactions lead to the results, we can provide feature importance rankings that explain which inputs are important for the AI-based CDSSs. Second level explainability allows us to identify which features were important for an individual prediction. Individual predictions can be safe-checked for patterns that might indicate a false prediction, e.g. in case of unusual feature distribution in an out-of-sample case. This second level explainability will regularly be available for AI-based CDSS but not for other diagnostic tests. This also has implications for the presentation of explainability results to doctors (and patients). Depending on the clinical use case and the risk attributed to that particular use case, first level explanations might be sufficient, whereas other use cases will regularly require second level explanations to safe-guard patients.

To date, clinical validation is currently the first widely discussed requirement for a medical AI system. Explainability is often only considered on second thought. The reason for this seems obvious: Medical AI systems and especially CDSSs, whether AI-powered or not, have to undergo a rigorous validation process to meet regulatory standards and achieve medical certification [1]. Once this process is completed successfully, there is proof that the system can perform in the highly heterogeneous real-world clinical setting. Here, it is important to understand how clinical validation is measured. A common performance indicator is prediction performance, often referred to as prediction accuracy. Different measures exist for prediction accuracy, tailored to certain use-cases, but their common characteristic is that they reflect the prediction quality and thus general clinical usefulness of a model. Thus, one of the main goals of model development is to increase prediction performance and provide low error rates. And, indeed, AI-powered systems have been shown to produce overall lower error rates than traditional methods [19–21].

Despite all efforts, however, AI systems cannot provide perfect accuracy owing to different sources of error. For one, because of naturally imperfect datasets in medicine (e.g. due to noise or recording errors), it is basically impossible to develop a model without *any* errors. These errors are random errors. Thus, there will always be certain cases of false positive and false negative predictions. For another, a particularly important source of error is AI bias. AI bias leads to systematic errors, a systematic deviation from the expected prediction behavior of the AI tool. Ideally, the data used for training fully represent the population in which the AI tool is later applied. A major goal of AI in healthcare product development is to approximate this ideal state via thorough clinical validation and development on heterogeneous data sources [1]. While this ensures that AI bias can be reduced to a minimum, it will still be almost impossible to generate AI tools without *any* trace of bias. If bias is present, then there will be prediction errors in patients not representing the training sample. Taken together, both random and systematic sum up to the total number of errors that physicians and patients will encounter in the clinical setting, even when a fully validated high-performing AI system is used.

This is why, from a medical point-of-view, not only clinical validation but also explainability plays an instrumental role in the clinical setting. Explainability enables the resolution of disagreement between an AI system and human experts, no matter on whose side the error in judgment is situated. It should be noted that this will succeed mostly in cases of systematic error, of AI bias, rather than in cases of random error. Random errors are much harder to identify and will likely go unnoticed in case of agreement between the tool and the physician or will lead to situations of disagreement between the tool and the physician. This situation is discussed in the ethical considerations section. Explainability results are usually represented visually or through natural language explanations. Both show the clinicians how different factors contributed to the final recommendation. In other words, explainability can assist clinicians in evaluating the recommendations provided by a system based on their experience and clinical judgment. This allows them to make an informed decision whether or not to rely on the system’s recommendations and can, consequently, strengthen their trust in the system. Particularly in cases where the CDSS produces recommendations that are strongly out of line with a clinician's expectations, explainability allows verification whether the parameters taken into account by the system make sense from a clinical point-of-view. By laying open the inner workings of the CDSS, explainability can, thus, assist clinicians in identifying false positives and false negatives more easily. As clinicians identify instances in which the system performs poorly, they can report these cases back to developers to foster quality assurance and product improvement. Given these considerations, explainability may be a key driver for the uptake of AI-driven CDSS in clinical practice, as trust in these systems is not yet established [22, 23]. Here, it is important to note that any use of AI-based CDSS may influence a physician in reaching a decision. It will, therefore, be of critical importance to establish transparent documentation on how recommendations were derived.

### The patient perspective

Looking at the issue of explainability from the patient perspective raises the question of whether the use of AI-powered decision aids is compatible with the inherent values of patient-centered care. Patient-centered care aims to be responsive to and respectful of individual patients’ values and needs [24]. It considers patients as active partners in the care process, emphasizing their right to choice and control over medical decisions. A key component of patient-centered care is shared decision-making aimed at identifying the treatment best suited to the individual patients’ situation [25, 26]. It involves an open conversation between the patient and the clinician, where the clinician informs the patient about the potential risks and benefits of available courses of action and the patient discusses their values and priorities [27, 28].

Several evidence-based tools have been developed to facilitate shared decision-making, among them, so-called conversation aids [29]. Unlike patient decision aids (which are used by the patient in preparation prior to the clinical encounter), conversation aids are designed for use within the clinical encounter to guide the patient and clinician through the shared decision-making process [28, 30]. They incorporate established medical facts about their conditions and, by synthesizing available information, they can help patients to understand their individual risks and outcomes, to explore the available options, and to determine which course of action best fits their goals and priorities [30–32]. So, what if individual risk was not calculated using established risk prediction models but instead relied on a validated, yet not explainable, data-driven approach? Would it make a difference from the patient’s perspective? Seeking to address these questions, it was recently argued that so-called ‘black-box medicine’ conflicts with core ideals of patient-centered medicine [33]. Since clinicians are no longer able to fully comprehend the inner workings and calculations of the decision aid they are not able to explain to the patient how certain outcomes or recommendations were derived [33].

Explainability can address this issue by providing clinicians and patients with a personalized conversation aid that is based on the patient’s individual characteristics and risk factors. By simulating the impact of different treatment or lifestyle interventions, an explainable AI decision aid could help to raise patients’ choice awareness and support clinicians in eliciting patient values and preferences [34]. As described previously, explainability provides a visual representation or natural language explanation of how different factors contributed to the final risk assessment. Yet, to interpret system-derived explanations and probabilities, patients rely on the clinician’s ability to understand and convey these explanations in a way that is accurate and understandable. If used appropriately, explainable AI decision support systems may not only contribute to patients feeling more knowledgeable and better informed but could also promote more accurate risk perceptions [34, 35]. This may, in turn, boost patients’ motivation to engage in shared decision-making and to act upon risk-relevant information [35].

### Ethical implications

With the increasing penetration of AI-powered systems in healthcare, there is a necessity to explore the ethical issues accompanying this imminent paradigm shift. A commonly applied and well-fitting ethical framework when assessing biomedical ethical challenges are the “Principles of Biomedical Ethics” by Beauchamp and Childress [36, 37] introducing four key principles: autonomy, beneficence, nonmaleficence, and justice [36]. While principlism is not the only available bioethical framework, it is a very useful basic practical framework with high acceptance both in research and medical settings [36–38]. Thus, in the following, we assess explainability with regards to the aforementioned four principles.

Concerning *autonomy*, explainability has implications for patients and physicians alike [31]. One of the major safeguards of patients’ autonomy is represented by informed consent, that is an autonomous, generally written authorization with which the patient grants a doctor his or her permission to perform a given medical act [39]. Proper informed consent is premised upon exhaustive and understandable information regarding the nature and risks of a medical procedure, and lack of undue interference with the patient’s voluntary decision to undergo the procedure. At the moment, an ethical consensus has not yet emerged as to whether disclosing the use of an opaque medical AI algorithm should be a mandatory requirement of informed consent. A failure to disclose the use of an opaque AI system may undermine patients’ autonomy and negatively impact the doctor-patient relationship, jeopardizing patients’ trust, and might violate the compliance with clinical recommendations. If the patient were to find out in hindsight that a clinician’s recommendation was derived from an opaque AI system, this may lead the patient to not only challenge the recommendation but might also lead to a justified request for explanation—which in the case of an opaque system, the clinician would not be able to provide. Opaque medical AI can, therefore, represent an obstacle to the provision of accurate information and thus potentially jeopardize informed consent. Appropriate ethical and explainability standards are therefore important to safeguard the autonomy-preserving function of informed consent.

Attention should be paid to the risk that the introduction of opaque AI into medical decision making may foster paternalism by limiting opportunities for patients to express their expectations and preferences regarding medical procedures [39]. A necessary prerequisite for shared decision making is full autonomy of the patient, but full autonomy can only be achieved if the patient is presented with a range of meaningful options to choose from [40]. In this respect, patients’ opportunities to exert their autonomy regarding medical procedures get reduced as opaque AI becomes more central to medical decision making. In particular, the challenge that arises with opaque CDSS is that it remains unclear whether and how patient values and preferences are accounted for by the model. This state of affairs could be addressed by means of “value-flexible” AI that provides different options for the patient [41]. We further argue that explainability is a necessary step towards value-flexible AI. The patient needs to be able to understand which variables play an important role in the inner workings of the AI system to determine—with the aid of the doctor—whether the goals and weighting of the AI system align with their values or not. For example, AI systems primed for “survival” as the outcome might not be aligned with the value of patients for whom a “reduction of suffering” is more important [41]. Lastly, when a choice is made, patients need to be able to trust an AI system to decide with confidence and autonomy to follow its guidance [42]. This is not possible when the AI model is opaque. Therefore, explainability is—both from the physician’s and patient’s point-of-view—an ethical prerequisite for systems supporting critical medical decision making.

While the principles of *beneficence* and *non-maleficence* are related, they nonetheless shed light on different aspects, also with regards to explainability. Beneficence urges physicians to maximize patient benefits. When applying AI-based systems, physicians are thus expected to use the tools in a manner that promotes the optimal outcome for the respective patient. Yet, to provide patients with the most appropriate options to promote their health and wellbeing, physicians need to be able to use the full capabilities of the system. This implies that physicians have knowledge of the system beyond a robotic application in a certain clinical use case, allowing them to reflect on the system’s output. For physicians, explainability in the form of visualizations or natural language explanations enables confident clinical decisions instead of having to simply trust an automated output. They can critically assess the system-derived outcomes and make their own judgments whether the results seem trustworthy or not. This allows them to adapt predictions and recommendations to individual circumstances where necessary. As such, clinicians can not only reduce the risk of eliciting false hope or creating false despair but can also flag potentially inappropriate interventions using their clinical judgment [43]. This is especially important when we imagine a situation where a physician and an AI system are in disagreement, a situation that is not easily resolved [42]. Fundamentally, this is a question of epistemic authority, and it is unclear how physicians should decide whether they can trust the epistemic authority of a black box model enough to defer to its decision [42]. Grote et al. [42] argue that in the case of opaque AI there is not enough epistemic support for deference. Moreover, they further argue that confronted with a black-box system, clinical decision support might not enhance the capabilities of physicians, but rather limit them. Here, physicians might be forced into “defensive medicine” where they dogmatically follow the output of the machine to avoid being questioned or held accountable [42]. Such a situation would cause a serious threat to physician autonomy. Additionally, physicians will rarely have the time to perform an in-depth analysis of why their clinical judgement is in disagreement with the AI system. Thus, looking merely at a performance output is not sufficient in the clinical context. The optimal outcome for all patients can only be expected with healthcare staff that can make informed decisions when to apply an AI-powered CDSS and how to interpret its results. It is thus hard to imagine how beneficence in the context of medical AI can be fulfilled with any “black box” application.

The need for explainability is also evident when assessing the principle of non-maleficence in the context of medical AI. Non-maleficence states that physicians have a fundamental duty not to harm their patients either intentionally or through excessive or inappropriate use of medical means. Why is performance not enough? It has been argued that a black box medical AI-based only on validated maximized performance is ethically justifiable even if the causal mechanisms behind a given AI-prescribed intervention remain opaque to the clinician [44]. Reliance on anecdotal or purely experiential evidence about the efficacy of a given treatment is indeed still quite common in medicine. Yet this is no excuse to forego explanations as a major requirement of sound clinical judgment when such an explanation is indeed possible. Recent progress in elucidating at least the principal features of AI models, while not providing full mechanistic explanations of AI-decisions, create a prima facie ethical obligation to reduce opacity and increase the interpretability of medical AI. Failure to do so would mean intentionally undermining a physician’s capacity to control for possible misclassifications of individual clinical cases due, for instance, to excessive bias or variance in training datasets. We thus conclude that also with regards to beneficence and non-maleficence, explainability is a necessary characteristic of clinically applied AI systems.

The principle of *justice* postulates that people should have equal access to the benefits of medical progress without ethically unjustified discrimination of any particular individuals or social group [36]. Some AI systems, however, violate this principle. Recently, for example, Obermeyer et al. reported on a medical AI system discriminating against people of color [5]. Explainability can support developers and clinicians to detect and correct such biases—a major potential source for injustice—ideally at the early stage of AI development and validation, e.g. by identification of important features indicating a bias in the model. However, for explainability to fulfill this purpose, the relevant stakeholder groups must be sensitized to the risk of bias and its potential consequences for individuals’ health and wellbeing. At times, it might be tempting to prioritize accuracy and simply refrain from investing resources into developing explainable AI. Yet to ensure that AI-powered decision support systems realize their potential, developers, and clinicians need to be attentive to the potential flaws and limitations of these new tools. Thus, also from the justice perspective, explainability becomes an ethical prerequisite for the development and application of AI-based clinical decision support.

## Conclusion

In this paper, we explored the role of explainable AI in clinical decision support systems from the technological, legal, medical, and patient perspectives. In doing so, we have shown that explainability is a multifaceted concept that has far-reaching implications for the various stakeholder groups involved. Medical AI poses challenges to developers, medical professionals, and legislators as it requires a reconsideration of roles and responsibilities. Based on our analysis, we consider explainability a necessary requirement to address these challenges in a sustainable manner that is compatible with professional norms and values.

Notably, a move towards opaque algorithms in CDSS may inadvertently lead to a revival of paternalistic concepts of care that relegate patients to passive spectators in the medical decision-making process. It might also bring forward a new type of medicine where physicians become slaves to the tool’s output to avoid legal and medical repercussions. And, last but not least, opaque systems might provoke a faulty allocation of resources violating their just distribution. In this paper, we have argued that explainability can help to ensure that patients remain at the center of care and that together with clinicians they can make informed and autonomous decisions about their health. Moreover, explainability can promote the just distribution of available resources.

We conclude that omitting explainability in clinical decision support systems poses a threat to core ethical values in medicine and may have detrimental consequences for individual and public health. Further work is needed to sensitize developers, healthcare professionals, and legislators to the challenges and limitations of opaque algorithms in medical AI and to foster multidisciplinary collaboration to tackle these challenges with joined forces.

## Data Availability

Not applicable.

## Abbreviations

AI: Artificial intelligence
ANN: Artificial neural network
CDSS: Clinical decision support system
FDA: Food and Drug Administration
GDPR: General Data Protection Regulation
ICU: Intensive care unit
MDR: Medical Device Regulation
TPLC: Total product lifecycle approach

## References

[1] Higgins D, Madai VI (2020). From bit to bedside: a practical framework for artificial intelligence product development in healthcare. Adv Intell Syst..

[2] Rudin C (2019). Stop explaining black box machine learning models for high stakes decisions and use interpretable models instead. Nat Mach Intell.

[3] Doran D, Schulz S, Besold TR. What does explainable AI really mean? A new conceptualization of perspectives. ArXiv171000794 Cs. 2017. http://arxiv.org/abs/1710.00794. Accessed 3 Sept 2019.

[4] Shortliffe EH, Sepúlveda MJ (2018). Clinical decision support in the era of artificial intelligence. JAMA.

[5] Obermeyer Z, Powers B, Vogeli C, Mullainathan S (2019). Dissecting racial bias in an algorithm used to manage the health of populations. Science.

[6] Samek W, Montavon G, Vedaldi A, Hansen LK, Müller K-R (2019). Explainable AI: interpreting, explaining and visualizing deep learning.

[7] Esteva A, Robicquet A, Ramsundar B, Kuleshov V, DePristo M, Chou K (2019). A guide to deep learning in healthcare. Nat Med.

[8] Islam SR, Eberle W, Ghafoor SK. Towards quantification of explainability in explainable artificial intelligence methods. ArXiv191110104 Cs Q-Fin. 2019. http://arxiv.org/abs/1911.10104. Accessed 2 Oct 2020.

[9] Samek W, Montavon G, Lapuschkin S, Anders CJ, Müller K-R. Toward interpretable machine learning: transparent deep neural networks and beyond. ArXiv200307631 Cs Stat. 2020. http://arxiv.org/abs/2003.07631. Accessed 2 Oct 2020.

[10] Lapuschkin S, Wäldchen S, Binder A, Montavon G, Samek W, Müller K-R (2019). Unmasking Clever Hans predictors and assessing what machines really learn. Nat Commun.

[11] Zech JR, Badgeley MA, Liu M, Costa AB, Titano JJ, Oermann EK (2018). Variable generalization performance of a deep learning model to detect pneumonia in chest radiographs: a cross-sectional study. PLOS Med.

[12] Olsen HP, Slosser JL, Hildebrandt TT, Wiesener C. What’s in the box? The legal requirement of explainability in computationally aided decision-making in public administration. SSRN Scholarly Paper. Rochester: Social Science Research Network; 2019. 10.2139/ssrn.3402974.

[13] Schönberger D (2019). Artificial intelligence in healthcare: a critical analysis of the legal and ethical implications. Int J Law Inf Technol.

[14] Cohen IG. Informed consent and medical artificial intelligence: what to tell the patient? SSRN Scholarly Paper. Rochester, NY: Social Science Research Network; 2020. 10.2139/ssrn.3529576.

[15] Beaudouin V, Bloch I, Bounie D, Clémençon S, d’Alché-Buc F, Eagan J (2020). Identifying the “right” level of explanation in a given situation. SSRN Electron J.

[16] FDA. Proposed regulatory framework for modifications to artificial intelligence/machine learning (AI/ML)-based Software as a Medical Device (SaMD). 2020. https://www.fda.gov/files/medical%20devices/published/US-FDA-Artificial-Intelligence-and-Machine-Learning-Discussion-Paper.pdf. Accessed 5 July 2020.

[17] Hacker P, Krestel R, Grundmann S, Naumann F. Explainable AI under contract and tort law: legal incentives and technical challenges. SSRN Scholarly Paper. Rochester, NY: Social Science Research Network; 2020. https://papers.ssrn.com/abstract=3513433. Accessed 13 Feb 2020.

[18] Ferretti A, Schneider M, Blasimme A (2018). Machine learning in medicine: opening the new data protection black box. Eur Data Prot Law Rev EDPL.

[19] Weng SF, Reps J, Kai J, Garibaldi JM, Qureshi N (2017). Can machine-learning improve cardiovascular risk prediction using routine clinical data?. PLoS ONE.

[20] Kakadiaris IA, Vrigkas M, Yen AA, Kuznetsova T, Budoff M, Naghavi M (2018). Machine learning outperforms ACC/AHA CVD risk calculator in MESA. J Am Heart Assoc..

[21] Liu T, Fan W, Wu C (2019). A hybrid machine learning approach to cerebral stroke prediction based on imbalanced medical dataset. Artif Intell Med..

[22] Cutillo CM, Sharma KR, Foschini L, Kundu S, Mackintosh M, Mandl KD (2020). Machine intelligence in healthcare—perspectives on trustworthiness, explainability, usability, and transparency. NPJ Digit Med.

[23] Tonekaboni S, Joshi S, McCradden MD, Goldenberg A. What clinicians want: contextualizing explainable machine learning for clinical end use. ArXiv190505134 Cs Stat. 2019. http://arxiv.org/abs/1905.05134. Accessed 3 Sept 2019.

[24] Institute of Medicine (US) Committee on Quality of Health Care in America. Crossing the quality chasm: a new health system for the 21st century. Washington, DC: National Academies Press (US); 2001. http://www.ncbi.nlm.nih.gov/books/NBK222274/. Accessed 21 May 2020. 25057539

[25] Barry MJ, Edgman-Levitan S (2012). Shared decision making—the pinnacle patient-centered care. N Engl J Med.

[26] Kunneman M, Montori VM, Castaneda-Guarderas A, Hess EP (2016). What is shared decision making? (and What it is not). Acad Emerg Med.

[27] O’Neill ES, Grande SW, Sherman A, Elwyn G, Coylewright M (2017). Availability of patient decision aids for stroke prevention in atrial fibrillation: a systematic review. Am Heart J.

[28] Noseworthy PA, Brito JP, Kunneman M, Hargraves IG, Zeballos-Palacios C, Montori VM (2019). Shared decision-making in atrial fibrillation: navigating complex issues in partnership with the patient. J Interv Card Electrophysiol.

[29] Dobler CC, Sanchez M, Gionfriddo MR, Alvarez-Villalobos NA, Ospina NS, Spencer-Bonilla G (2019). Impact of decision aids used during clinical encounters on clinician outcomes and consultation length: a systematic review. BMJ Qual Saf.

[30] Noseworthy PA, Kaufman ES, Chen LY, Chung MK, Elkind Mitchell SV, Joglar JA (2019). Subclinical and device-detected atrial fibrillation: pondering the knowledge gap: a scientific statement from the American Heart Association. Circulation.

[31] Spencer-Bonilla G, Thota A, Organick P, Ponce OJ, Kunneman M, Giblon R (2020). Normalization of a conversation tool to promote shared decision making about anticoagulation in patients with atrial fibrillation within a practical randomized trial of its effectiveness: a cross-sectional study. Trials.

[32] Bonner C, Bell K, Jansen J, Glasziou P, Irwig L, Doust J (2018). Should heart age calculators be used alongside absolute cardiovascular disease risk assessment?. BMC Cardiovasc Disord.

[33] Bjerring JC, Busch J (2020). Artificial intelligence and patient-centered decision-making. Philos Technol.

[34] Politi MC, Dizon DS, Frosch DL, Kuzemchak MD, Stiggelbout AM (2013). Importance of clarifying patients’ desired role in shared decision making to match their level of engagement with their preferences. BMJ.

[35] Stacey D, Légaré F, Lewis K, Barry MJ, Bennett CL, Eden KB (2017). Decision aids for people facing health treatment or screening decisions. Cochrane Database Syst Rev.

[36] Beauchamp TL. Principles of biomedical ethics. Paperback May-2008. New York: Oxford University Press; 2008.

[37] Gillon R (2015). Defending the four principles approach as a good basis for good medical practice and therefore for good medical ethics. J Med Ethics.

[38] Mittelstadt B (2019). Principles alone cannot guarantee ethical AI. Nat Mach Intell.

[39] Faden RR, Beauchamp TL (1986). A history and theory of informed consent.

[40] Raz J (2020). The Morality of Freedom.

[41] McDougall RJ (2019). Computer knows best? The need for value-flexibility in medical AI. J Med Ethics.

[42] Grote T, Berens P (2019). On the ethics of algorithmic decision-making in healthcare. J Med Ethics.

[43] Beil M, Proft I, van Heerden D, Sviri S, van Heerden PV (2019). Ethical considerations about artificial intelligence for prognostication in intensive care. Intensive Care Med Exp.

[44] London AJ (2019). Artificial intelligence and black-box medical decisions: accuracy versus explainability. Hastings Cent Rep.

